# Sex Differences in Factors Affecting Hospital Outpatient Department Visits: Korea Health Panel Survey Data from 2009 to 2016 †

**DOI:** 10.3390/ijerph16245028

**Published:** 2019-12-10

**Authors:** Chang Hoon You, Young Dae Kwon, Sungwook Kang

**Affiliations:** 1 Graduate School of Public Health, Yonsei University, Seoul 03722, Korea; chyou@yuhs.ac; 2 Department of Humanities and Social Medicine, College of Medicine and Catholic Institute for Healthcare Management, the Catholic University of Korea, Seoul 06591, Korea; 3 Department of Public Health, Daegu Haany University, Gyeongsan 38610, Korea; health@dhu.ac.kr

**Keywords:** sex differences, outpatient visits, socioeconomic status, sex inequality

## Abstract

This study intends to inspect the sex differences in proportion of hospital outpatient department (OPD) visits in overall outpatient (OP) visits using national panel data and to explore factors that influence the proportions by sex. This study analyzed data of the 2009–2016 Korea Health Panel Survey. Fractional logit regression was applied to analyze factors that affect proportion of hospital visits among outpatient visits. Analysis of related factors was carried out first for all analysis subjects and then by sex. The study data were provided by 7470 women (52.2%) and 6846 men (47.8%). The overall average number of OP visits was 13.0, and women showed a much higher frequency of visits (15.8) than men (9.9). The average proportion of hospital OPD visits among overall OP visits was 21.9%, and men showed a higher rate (25.1%) than women (19.5%). The analysis model including sociodemographic factors, economic factors, and health-related factors confirmed that men showed a higher rate of hospital usage than women. Type of medical security, household income, participation in economic activities, disability, and serious illnesses were significant variables for both sexes. Age, education level, marital status, and subscription to voluntary private health insurance were significant only for women, whereas region of residence was significant only for men. This study confirmed that there is a sex difference in proportion of hospital OPD visits and in the factors that affect the proportion of hospital OPD visits. Universal health coverage is provided through social health insurance, but there is a sex difference in hospital OPD visits, and factors related to socioeconomic status have a significant effect, especially on women’s selection of health care institutions. More attention should be given to sex differences in factors affecting health care utilization.

## 1. Introduction

Women have a longer life expectancy than men but utilize more health care services due to high morbidity [1,2,3,4,5]. However, not all studies show consistent results in sex differences in health care utilization, as results differ depending on type of health care service. Women use more prophylactic and examination services, while men use more emergency medicine or inpatient treatment services [6,7,8,9,10,11].

There have been many studies on the reasons for sex differences in health care utilization, but the results are inconsistent. Previous studies have defined factors related to reproductive health, such as pregnancy and childbirth [12,13,14], the higher morbidity in women than men, the sex difference in recognizing or reporting a disease or symptom [1,12,15,16], and the existence of a sex difference in the degree to which a person demands help or information related to a disease or its prevention [1,3,17]. Gender differences in the impact of sociocultural factors on health care utilization have also been reported [18,19,20]. Culturally, women in Korea are in many cases fully responsible for the household work for their parents, husbands, and children, and spend most of their time on raising their children. Under these cultural circumstances, women are likely to deepen the gender gap in health care utilization, with relatively large neglect of spending or investment on their own health care compared to other family members. As a result, women have been reported to have more medically unmet needs compared to men [21,22]. 

Among the need, enabling, or predisposing factors that affect health care utilization, previous studies mainly focused on need and predisposing factors, with few studies researching enabling factors. Enabling factors are those that make possible health care utilization and can be divided into family resources, such as income, property, and insurance; and community resources, such as medical resources, time needed to access health care institutions, and time spent waiting to receive health services [23]. Some studies reported that differences in the socioeconomic status such as income, economic level, and social standing can affect the sex difference in health care utilization. However, since most of these studies were carried out in relation to developing or underdeveloped countries, it is difficult to directly apply these results to developed countries [24,25,26]. In particular, in a situation of increasing financial burden of health care utilization due to active introduction of new medical technologies or drugs, it can be assumed that social and private health insurances have a large effect on health care utilization. This, in turn, affects the sex differences in health care utilization, although related studies are limited. 

Health insurance has an effect not only on quantity of health care utilization, but also on its quality, including selection of health care providers and health care institutions [27,28,29,30,31,32,33]. Rand Health Insurance Experiments are well known for empirical analytical study based on insurance theory and show that health insurance policyholders with a low cost share used more of both outpatient and inpatient services [27,28]. In studies of various European countries, in cases of subscription to voluntary health insurances, visits to specialists increased compared to those to general practitioners [25,27], and a study of diabetes patients reported that patients with supplementary private health insurance visited hospitals more than physician offices [33].

The health care delivery system in South Korea has a three-tier provision system in place that consists of primary, secondary, and tertiary health facilities. Patients are free to choose any primary and secondary levels of medical institutions for consultation, diagnosis, or treatment. However, patients’ copayment is higher when they get care from the secondary care facilities rather than the primary care facilities. Tertiary care facilities can be accessed only with a referral from primary or secondary care facilities except for emergencies, childbirth, etc. [34]. Although hospital outpatient department (OPD) use is more economically burdensome than primary care facility use, many patients prefer hospital OPD use. This is because hospital outpatient care is considered to have advantages in terms of quality care such as receiving some kinds of care needed at one visit, better equipped facilities and medical equipment, and being directly linked to surgical treatment or inpatient care [35,36]. 

This study intends to inspect the sex difference in proportion of hospital OPD visits for overall outpatient (OP) visits using national panel data and to explore factors that influence the proportion of hospital OPD visits in overall OP visits by sex.

## 2. Methods

### 2.1. Data and Subjects

This study analyzed the 2009–2016 data of the Korea Health Panel Survey (KHPS), a government-approved statistical survey carried out under joint supervision of the Korea Institute for Health and Social Affairs and the National Health Insurance Service. This survey has been carried out every year since 2008, and examines not only the amount of health care utilization, including hospitalization, outpatient visits, medication, and medical expenditures, but also main behaviors related to health care utilization and financial resources such as voluntary health insurance. Sampling was performed with a stratified method using 90% of the 2005 census data and comprised approximately 8000 households from 350 survey areas. Survey items for households included the number of household members, income, and expenditure, and survey items for household members included sociodemographic and economic characteristics, as well as health service utilization, health status, and health behaviors, collected through a self-report method. 

In the present study, yearly data on health care utilization, health behavior, and voluntary insurance subscription from the Korea Health Panel Survey from 2009 to 2016 was integrated with individual sociodemographic characteristics to compile panel data for analysis. Among all survey participants, adults aged 20 years and above were selected as study subjects. As of 2009, the total number of subjects was 14,316, comprising 7470 men and 6846 women. 

This study was approved by the Institutional Review Board of The Catholic University of Korea (MC19ZESI0002) with a waiver for informed consent because the data were obtained from a public data depository that is freely accessible online (https://www.khp.re.kr:444/). 

### 2.2. Variables and Measurement

The proportion of secondary or tertiary hospital OPD visits among overall OP visits by individual subjects in each given year was selected as the dependent variable. The KHPS examines in detail each individual case of health care utilization, including hospitalization and outpatient visits. For outpatient visits, the survey examines detailed information about visits, such as date and time, diagnosis, treatment expenditure, diagnostic examination, and type of health care institution. Health care institutions are divided into primary, secondary, and tertiary institutions; the present study used the ratio of visits to secondary and tertiary institutions only as the dependent variable. 

The explanatory variables were selected by referring to previous studies that examined factors that affect health care utilization and were broadly divided into sociodemographic, economic, and health characteristics. The sociodemographic characteristics were age, education level, marital status, region of residence, type of medical security, and subscription to supplementary private health insurances. In cases of voluntary private health insurances, only indemnity plans were included. Age groups were divided into ‘20–44 years’, ‘45–64 years’, ‘65–79 years’, and ‘80 years and above’. Education level was divided into ‘elementary school or under,’ ‘middle school or high school graduate,’ and ‘college graduate and above.’ Region of residence was divided into ‘metro regions’ of Seoul, Gyeonggi Province, and Incheon and ‘other regions’. Type of medical security was divided into Medical Aid and National Health Insurance. Economic characteristics were household income per capita converted into a logarithmic value for analysis and economic activity. Health characteristics included unmet need for health care, disability, cancer, and cardiovascular diseases. A ‘yes’ answer to the question “During the past year, have needed hospital treatment or examination, but were unable to obtain it?” was considered as unmet need for health care. Disability, cancer, and cardiovascular diseases were considered only if they were diagnosed by a doctor. 

### 2.3. Statistical Analysis

In order to provide sample characteristics, a set of descriptive analyses were conducted. The frequency, percentage, and mean and standard deviation of the sample were calculated. Bivariate analysis, either a t or chi-squared test according to variable type, was performed to determine the difference in distribution of characteristics between sexes. Then, fractional logistic regression was performed to analyze the factors that affect proportion of hospital OPD visits among overall OP visits. Fractional logistic regression is one of the models used to analyze ratio data as analysis of ratio data with a general regression model violates the basic assumption of linear regression and can involve significant bias in the estimated regression coefficients. To resolve this problem, Papke and Woodridge (1996) proposed a fractional logit model that allows ratio data to be analyzed with inclusion of the case where the response variable Y_i_ is 0 ≤ Y_i_ ≤ 1, i.e., the response variable is observed between specific discrete values 0 and 1, using quasi- likelihood estimation [37]. This study used the fractional logit model to analyze the factors related to proportion of hospital OPD visits among overall OP visits. Analysis of related factors was carried out first for all analysis subjects and then by sex. Statistical analysis was performed with Stata ver. 14 (StataCorp LLC, College Station, TX, USA).

## 3. Results

The bassline characteristics of the 14,316 study subjects as of 2009 are shown in Table 1. The 7470 women (52.2%) and 6846 men (47.8%) had an average age of 48.2 years, and the women’s average age was higher (48.8 years) than that of men (47.6 years). The overall education of men was higher than that of women—43.0% of men had an education level of college graduate or higher, whereas 29.9% of women had completed higher education. The proportion of married subjects was larger among men (73.6%) than among women (67.4%). The household income per capita was higher among men (37.348 million Korean won) than among women (35.185 million Korean won). More men were economically active (74.0%) than women (47.6%). 

Experience with unmet medical needs was higher among women (21.9%) than among men (15.5%). The disability rate was higher among men (6.8%) than among women (4.9%). The morbidity rate of cancer was higher among men (29.3%) than among women (12.4%), whereas the morbidity rate of cardiovascular diseases was higher among women (7.3%) than among men (5.4%). The overall average number of outpatient visits was 13.0, and women showed a much higher frequency of visits (15.8) than men (9.9). Total outpatient expenditures were greater among women (299,000 Korean won) than among men (178,000 Korean won). The average proportion of hospital OPD visits among overall OP visits was 21.9%, and men showed a higher rate (25.1%) than women (19.5%; Table 1).

Table 2 shows the results of the multivariate analysis using a fractional logit model in relation to factors that affect proportion of hospital OPD visits among overall OP visits. According to the analysis results of all subjects, sex, age, education level, marital status, region of residence, type of medical security, voluntary private health insurance, household income, disability, cancer, and cardiovascular diseases were significant variables. There was a higher ratio of hospital OPD visits among the following: men, middle age (45–64 years), higher education level (middle school/high school graduate, college graduate and above), voluntary private health insurance subscribers, higher household income, disability, cancer, and cardiovascular disease. On the other hand, people who were divorced/separated by death, capital region dwellers, National Health Insurance subscribers, and economically active people showed a lower ratio of hospital OPD visits (Table 2). 

According to the results of analysis of only women, the following are linked to a higher ratio of hospital OPD visits to overall OP visits: middle age (45–64 years), higher education (middle school/high school graduate, college graduate and above), voluntary health insurance subscriber, higher household income, disability, cancer, and cardiovascular disease. On the other hand, those who were divorced/separated by death or unmarried, subscribed to the National Health Insurance, and those who were economically active showed a lower ratio of hospital OPD visits. 

According to analysis of only men, higher household income, disability, cancer, and cardiovascular disease were related to a higher ratio of hospital OPD visits to overall OP visits. On the other hand, capital region dwellers, National Health Insurance subscribers, and economically active individuals showed a lower ratio of hospital OPD visits (Table 3). 

## 4. Discussion

This study analyzed factors that influence proportion of hospital OPD visits among overall OP visits using the 2009–2016 data from the KHPS. Men showed a higher ratio of hospital visits compared to women, and the total amount of outpatient service usage (amount of outpatient visits and outpatient expenditures) was larger among women than among men, while men showed a higher proportion of hospital visits. The analysis model including sociodemographic, economic, and health-related factors confirmed that men showed a higher rate of hospital usage than women, while women perform more outpatient visits than men. Many study results show this sex difference in outpatient visits [1,2,15,38,39]. 

However, because not many studies have analyzed selection of health care institutions (health care providers) or amount of health care utilization based on sex differences, it is difficult to directly compare the results among studies. Many studies considered sex as a control variable in the analysis process. Cooper (1996) grouped analysis subjects by sex and whether they had reached 65 years of age and analyzed the factors of selecting specialists; the influence of sex was not clear [40]. McGlone (2002) executed a factor analysis and concluded that the sex effect on physician selection was not considerable, but the analysis was not based on actual health care utilization [41]. Buchmueller (2004) stated that men were less likely than women to choose specialists over general practitioners in France, but the difference in health delivery systems between countries should be taken into account [30]. You (2018) stated that sex difference was not significant in the proportion of hospital outpatient visits in relation to clinic visits, but the study was limited to diabetes patients [33].

Women tend to utilize health care when they experience light symptoms or problems in the early stage of a disease, whereas men are more likely to turn to health care institutions only after they experience severe symptoms or a serious condition [1,3,15,42]. This sex difference in behavior might be one of the reasons why women utilize outpatient services more than men as well as a reason why men have a higher rate of hospital OPD visits to overall OP visits. The high rate of hospital OPD visit may indicate that the need for multidisciplinary treatment or advanced examination is relatively high due to the high severity of the disease. Considering this sex difference in behavior, this study included morbidity from cancer and cardiovascular diseases, which are the most common serious diseases, in the analysis model. However, it cannot be guaranteed that this sufficiently controlled the seriousness or severity of a disease. 

This study confirmed sex difference in factors that influence the proportion of hospital OPD visits among overall OP visits. Type of medical security, household income, participation in economic activities, disability, and serious illnesses were significant variables for both sexes. Age, education level, marital status, and subscription to voluntary health insurance were significant only for women, whereas region of residence was significant only for men. 

In South Korea, health care users can freely choose health care institutions regardless of its type for outpatient visits, and economic factors (since treatment at hospitals is more expensive), distance, and time affect selection of health care institution [43,44,45,46]. Andersen’s health care utilization model considers these as enabling factors. In the present study, variables closely related to socioeconomic status significantly affected women’s choice of health care institutions. Women with a higher level of education showed a higher rate of hospital visits, while women who lived alone showed a lower rate of hospital visits. Women who subscribed to voluntary health insurance showed a higher rate of hospital visits. In South Korea, all citizens are covered by the National Health Insurance, but there is a considerably high out-of-pocket burden. To alleviate this financial burden, many subscribe to supplementary private health insurance [47]. In the present study, the effect of voluntary private health insurance was significant only for women. 

While there was no sex difference in need factors such as disability and illness, there was a clear difference among women in selecting health care institutions depending on education level, economic level, and household type. Many previous studies have demonstrated that socioeconomic status affects women more than men in overall health care utilization [2,26,48,49]. The present study confirmed that, in health care utilization behavior including not only health care usage, but also selection of health care institutions, factors related to socioeconomic status have a more significant influence on women than men. 

In the northeast Asian Confucian culture including Korea and China, men possess superior positions over women in all areas of society, which leads to sex disparities or inequalities not only in education and employment, but also in economic rights and distribution of inheritance [10,50,51]. Inequalities in education or employment made it difficult for women to achieve economic independence, and this effect was exacerbated by the marriage system. This inequality resulted in men making most financial decisions and lack of concern regarding needs of women. This sex inequality increases with lower socioeconomic status [52,53,54]. Rapid economic development and improvement of socio-political systems since the late 20th century have significantly reduced sex inequalities, but this study confirmed clear sex differences in health care utilization behavior, assumed to be a result of these difference in the effect of socioeconomic status on health care utilization. 

This study examined outpatient health care utilization of study subjects as clinic visits and hospital visits and used the ratio of hospital visits to all outpatient visits as the dependent variable. Generally, if the dependent variable is a ratio variable between 0 and 1, linear regression is used for analysis. However, when using linear regression, if the estimated value is less than 0 or greater than 1, it does not satisfy the consistency assumption and allow high possibility of bias in the analysis process. The beta regression analysis proposed by Ferrari and Crubaru-Neto (2004) and Smithson and Verkuilen (2006) is used to apply ratio analysis to dependent variables, but it is generally difficult to make estimations if the ratio value is a boundary value of 0 or 1 [55,56]. In health care utilization, there are cases of outpatient visits where patients would only use clinic-level or hospital-level institutions depending on condition severity, greatly limiting the applicability of this method. Thus, the present study applied the expanded fractional logit regression method [57], originally proposed by Papke and Wooldrige (1996), to the panel data. Few previous studies used this method in health service research, which may be because existing public panel data are usually aggregate data. The Korea Health Panel Survey data provides information about health care utilization for each outpatient visit, allowing use of the ratio regression analysis method. This use allowed analysis from a different point of view compared to previous analysis using aggregate data. 

While this study focused on the characteristics of health care users, the characteristics of health care providers or institutions can also affect selection of health care institutions [58]. Although availability of medical equipment or well-known staff can affect patient choice of health care institutions, this was not considered in this study due to limited source data. Depending on health care institution characteristics, it is possible to undergo surgery or receive treatment on the same day as an outpatient visit, which can influence outpatient service utilization; however, this was not considered in the present study. This study assessed the health status of subjects based on objective indicators, such as disability, oncological diseases, and cardiovascular diseases. There is a possibility that self-rated health may also have an effect, but it was not aptly considered in this study, which is a limitation. In this study, there was a lack of information about the beneficiaries of programs or policies related to health care utilization. Therefore, this may lead to bias in some outcomes because of the lack of proper controlling variables in the model. In the future, there should be an expansion of analysis models or an improvement of analysis methods to reduce these limitations. 

## 5. Conclusions

This study confirmed a sex difference in proportion of hospital OPD visits and in factors that affect proportion of hospital OPD visits. Universal health coverage is provided through social health insurance, but there is a sex difference in hospital OPD visits, in which factors related to socioeconomic status have a significant effect, especially on women’s selection of health care institutions. In future studies and policies related to health care delivery systems or medical security, more attention should be given to sex differences in factors affecting health care utilization. 

## Figures and Tables

**Table 1:** Characteristics of the study population in 2009 (*n* = 14,316).

Variable		Female		Male		Total		Chi/t	*p*-Value
		*n*	%	*n*	%	*n*	%		
Age (years, mean ± SD)		48.8 ± 16.7		47.6 ± 15.9		48.2 ± 16.3		4.40	<0.001
Age group (years)								35.51	<0.001
	20–44	3354	44.9	3190	46.6	6544	45.7		
	45–64	2507	33.6	2410	35.2	4917	34.4		
	65–79	1387	18.6	1124	16.4	2511	17.5		
	≥ 80	222	3.0	122	1.8	344	2.4		
Education								523.71	<0.001
	Elementary school	2087	27.9	940	13.7	3027	21.1		
	Middle/high school	3147	42.1	2962	43.3	6109	42.7		
	College	2236	29.9	2944	43.0	5180	36.2		
Marital status								637.81	<0.001
	Married	5030	67.4	5017	73.6	10,047	70.4		
	Divorced/separated	1308	17.5	312	4.6	1620	11.3		
	Never married	1121	15.0	1492	21.9	2613	18.3		
Residence								0.71	0.401
	Metropolitan area	3119	41.8	2906	42.5	6025	42.1		
	Others	4351	58.3	3940	57.6	8291	57.9		
Health insurance								11.70	<0.001
	Medical Aid	326	4.4	221	3.3	547	3.9		
	National Health Insurance	7124	95.6	6537	96.7	13,661	96.2		
Private health insurance								2.47	0.116
	Yes	795	10.6	674	9.9	1469	10.3		
	No	6675	89.4	6172	90.2	12,847	89.7		
Household income per capita (10,000 KRW, mean ± SD)								−3.24	0.001
		3518.5 ± 2904.3		3734.8 ± 2861.7		3653.2 ± 2884.9			
Economic activity								1034.82	<0.001
	Yes	3559	47.6	5065	74.0	8624	60.2		
	No	3911	52.4	1781	26.0	5692	39.8		
Unmet need for healthcare								90.38	<0.001
	Yes	1589	21.9	977	15.5	2566	19.0		
	No	5652	78.1	5317	84.5	10,969	81.0		
Disability								24.08	<0.001
	Yes	366	4.9	467	6.8	833	5.8		
	No	7104	95.1	6379	93.2	13,483	94.2		
Cancer								622.73	<0.001
	Yes	928	12.4	2004	29.3	2932	20.5		
	No	6542	87.6	4842	70.7	11,384	79.5		
Cardiovascular disease								22.28	<0.001
	Yes	547	7.3	369	5.4	916	6.4		
	No	6923	92.7	6477	94.6	13,400	93.6		
No. of outpatient visits per year (mean ± SD)		15.8 ± 21.9		9.9 ± 19.9		13.0 ± 21.1		16.86	<0.001
Outpatient expenses (10,000 KRW, mean ± SD)		29.9 ± 69.0		17.8 ± 52.4		24.1 ± 61.9		11.74	<0.001
Proportion of hospital visits among all outpatient visits (%, mean ± SD)		19.5 ± 29.7		25.1 ± 35.7		21.9 ± 32.5		−9.07	<0.001
Total		7470	52.2	6846	47.8	14,316	100.0		

SD, standard deviation; KRW, Korean won.

**Table 2:** Factors affecting proportion of hospital outpatient department visits among overall outpatient visits.

Variable		Coefficient	S.E.	*p*-Value
Sex (ref = female)		0.2127	0.0205	< 0.001
Age group (ref = 20–44; years)				
	45–64	0.0581	0.0214	0.007
	65–79	−0.0283	0.0292	0.333
	≥80	0.0275	0.0415	0.507
Education (ref = elementary school)				
	Middle/high school	0.0920	0.0287	0.001
	College	0.1141	0.0347	0.001
Marital status (ref = married)				
	Divorced/separated	−0.0634	0.0300	0.035
	Never married	−0.0103	0.0320	0.747
Residence (ref = others)		−0.0538	0.0198	0.007
Health insurance (ref = Medical Aid)		−0.2702	0.0421	< 0.001
Private health insurance (ref = no)		0.0831	0.0181	< 0.001
Household income per capita		0.0767	0.0110	< 0.001
Economic activity (ref = no)		−0.0985	0.0157	< 0.001
Unmet need for healthcare (ref = no)		−0.0243	0.0146	0.097
Disability (ref = no)		0.3392	0.0325	< 0.001
Cancer (ref = no)		0.1886	0.0138	< 0.001
Cardiovascular disease (ref = no)		0.1976	0.0159	< 0.001
Intercept		−2.1041	0.0907	< 0.001
No. of observations		70,936		
Wald *(p*-value)		712.34 (*p* < 0.001)		

S.E., standard error; ref, reference.

**Table 3:** Factors affecting proportion of hospital outpatient department visits among overall outpatient visits by sex.

Variable		Female			Male		
		Coefficient	S.E.	*p*-Value	Coefficient	S.E.	*p*-Value
Age group (ref = 20–44; years)							
	45–64	0.0670	0.0290	0.021	0.0546	0.0322	0.089
	65–79	−0.0009	0.0407	0.982	−0.0370	0.0430	0.389
	≥ 80	0.0934	0.0584	0.110	−0.0145	0.0602	0.810
Education (ref = elementary school)							
	Middle/high school	0.0965	0.0381	0.011	0.0672	0.0445	0.131
	College	0.1686	0.0486	0.001	0.0680	0.0504	0.177
Marital status (ref = married)							
	Divorced/separated	−0.1172	0.0360	0.001	0.0909	0.0582	0.118
	Never married	−0.1361	0.0463	0.003	0.0788	0.0461	0.087
Residence (ref = others)		−0.0427	0.0267	0.110	−0.0618	0.0296	0.037
Health insurance (ref = Medical Aid)		−0.2340	0.0560	< 0.001	−0.3047	0.0647	< 0.001
Private health insurance (ref = no)		0.1054	0.0233	< 0.001	0.0543	0.0286	0.058
Household income per capita		0.0764	0.0147	< 0.001	0.0770	0.0169	< 0.001
Economic activity (ref = no)		−0.0684	0.0193	< 0.001	−0.1242	0.0272	< 0.001
Unmet need for healthcare (ref = no)		−0.0290	0.0190	0.127	−0.0198	0.0229	0.389
Disability (ref = no)		0.3052	0.0494	< 0.001	0.3477	0.0435	< 0.001
Cancer (ref = no)		0.2001	0.0178	< 0.001	0.1761	0.0219	< 0.001
Cardiovascular disease (ref = no)		0.1861	0.0216	< 0.001	0.2105	0.0235	< 0.001
Intercept		−2.1613	0.1196	< 0.001	−1.8224	0.1383	< 0.001
No. of observations		40,662			30,314		
Wald (*p*-value)		324.42 (*p* < 0.001)			299.28 (*p* < 0.001)		

S.E., standard error; ref, reference.
